# The effect of remnant forest on insect successional response in tropical fire-impacted peatland: A bi-taxa comparison

**DOI:** 10.1371/journal.pone.0174388

**Published:** 2017-03-23

**Authors:** Kok-Boon Neoh, Lee-Jin Bong, Ahmad Muhammad, Masayuki Itoh, Osamu Kozan, Yoko Takematsu, Tsuyoshi Yoshimura

**Affiliations:** 1 Department of Entomology, National Chung Hsing University, Taichung, Taiwan; 2 Laboratory of Innovative Humano-Habitability, Research Institute for Sustainable Humanosphere, Kyoto University, Gokasho, Uji, Kyoto, Japan; 3 Department of Biology, Faculty of Mathematics & Natural Sciences, Riau University, Pekanbaru, Indonesia; 4 Center for Southeast Asian Studies, Kyoto University, Kyoto, Japan; 5 Faculty of Agriculture, Yamaguchi University, Yamaguchi, Japan; Centre for Cellular and Molecular Biology, INDIA

## Abstract

Fire has become a common feature in tropical drained peatlands, and it may have detrimental impacts on the overall biodiversity of the forest ecosystem. We investigated the effect of fire on termite and ant assemblages and the importance of remnant forest in restoring species diversity in fire-impacted tropical peat swamp forests. The species loss of both termites and ants was as high as 50% in some fire-impacted peats compared to remnant forests, but in most cases the species richness for termites and ants was statistically equal along the land uses surveyed. However, a pronounced difference in functional group composition of termites was detected. In particular, sites close to remnant forests contained two additional termite feeding groups so that they shared a similar composition structure with remnant forests but were significantly different from sites distant from remnant forests. In general, ants were resilient to fire, and the similarity index showed a high degree of similarity among ant communities in all land uses surveyed. The Shannon diversity index for termites and ants decreased with increasing distance from the remnant forests and level of ecological degradation. Peat vegetation variables and ecological degradation were important in shaping termite and ant communities in the tropical peatlands, but their relative importance was not significant in fire-impacted peats regardless of distance from the remnant forests. This study highlights the importance of remnant forests as a biodiversity repository and natural buffer that can enhance species diversity and recolonization of forest-adapted species.

## Introduction

The forest cover of peat swamps in the Indo-Malayan region has decreased by 41% since the early 1990s and contributed only 36% of the total forest area in 2010. If the current deforestation rate progresses at 4.9% per year, Southeast Asian peat swamp forests are likely to disappear off the face of the Earth in the next two decades [1]. Tropical peat swamp forests, which are sensitive to disturbance, have been subjected to logging for valuable timber and land conversion for agro-industrial plantations. As a result, fire has become a common feature in the drained peatlands due to the highly flammable nature of dry peat. In addition, the El Nino phenomenon has intensified the fires in recent decades [2].

In temperate peat bogs, highly mosaic peat swamp forests have the potential to support a considerable number of arthropods, which are vital to ecosystem function [3,4,5,6]. They also serve as bioindicators of the peat recovery process [7,8]. In tropical forest ecosystems, termites and ants are highly abundant and diverse insect groups. Termites play pronounced roles as soil ecosystem engineers that help with soil fertilization, decomposition, and nutrient-carbon cycling; while ants act as seed dispersers, mutualists, and direct predators on multiple taxa, and they assist in soil processing and nutrient cycling [9,10]. Recent studies of the food web at multiple trophic levels in a tropical rain forest suggested that predators (e.g., ants) are highly dependent on herbivores and detritivores (e.g., termites) as part of their diet [11,12]. The feeding natures of termites and ants suggest that they may be fundamental in food web dynamics in a given tropical peat swamp forest and may help maintain the stability of the ecological system. Termite and ant diversity usually decreases with increased land use disturbance [13,14,15]. The relatively high temperature of tropical peat fires and their long-lasting smoldering effects elicit greater detrimental impacts on soil-dwelling insects (e.g., termites) [16].

Taxa lost in fire-impacted peatlands and in oil palm plantations converted from peatlands may be able to recover through successional processes, mainly via two possible mechanisms. First, the recovery of forest structure and the availability of food sources after a fire are the prime ecological drivers for succession. For example, the termite assemblage in fire-impacted wet savanna was found to be was highly diverse, likely because of the rich vegetation structure post-fire [17,18]. For ants, leaf litter volume, forest structure, and vegetation structure and composition were significant determinants shaping the leaf litter ant community structure in the Amazonian forest and savanna [19,20]. The dominance of opportunist ants in a central North American grassland was associated with increased plant biomass [21]. Second, remnant forests may act as sources to restore the loss of functionally important species in fire-impacted peatlands. Seasonal water inundation of the forest floor and the acidic nature of peat have led to the misconception that tropical peat swamps support low biodiversity and abundance, thus fewer in-depth biodiversity studies of this ecosystem have been conducted compared to temperate zonal peat bogs [22]. However, the biodiversity of peat swamps, including plants, mammals, birds, reptiles, amphibians, and fishes, is now receiving increasing attention from biologists, and a recent review reported that tropical peat swamp forests support numerous rare and threatened species [23]. Thus, these remnant forests or protected areas are seen as biodiversity reservoirs that are necessary for succession in fire-impacted land use areas. The influence of remnant forests on vertebrate and vegetation succession in human-modified landscapes is obvious [24], but no clear pattern for arthropods has been reported [25,26]. Limited evidence of arthropod succession in a tropical oil palm plantation due to the effect of forests is available for butterflies [27], ants [28], and orchid bees in a neotropical oil palm landscape [29]. However, the degree to which both mechanisms (i.e., recovery of habitat complexity post-fire and remnant forests) could act as main drivers to restore the biodiversity loss in fire-impacted peatland remains poorly understood.

The goals of this study were to address the following questions: Is there any difference in functional diversity and community structure of the studied taxa between fire-impacted peats far from the remnant forest and those close to the remnant forests compared with the assemblage in the remnant forests? and is the effect of environmental variables or the effect of remnant forests more important in supporting termite and ant diversity and shaping their community structure? We studied three sites within remnant forests, six sites that were close to remnant forests, and five sites that were within 10 km from the nearest forest in the same region. This allowed us to exclude any taxa structure discrepancies at the regional scale and minimize the effect of habitat heterogeneity.

## Materials and methods

### Study sites

We conducted the study in the Giam Siak Kecil–Bukit Batu Biosphere Reserve (0°44'–1°11'N, 0°11'–102°10'E) in Riau Province, Indonesia. This research was undertaken with the field permit number 412/SIP/FRP/SM/X/2012 from Ministry of Research, Technology and Higher Education of the Republic of Indonesia. Fires in the croplands have been prevalent following decades-long ground water drainage. Different management practices of the fire-impacted peatlands after fire have created a structurally heterogeneous landscape with fragments of abandoned peat forests, cultivated lands, and remnant forests.

**Fig 1:**
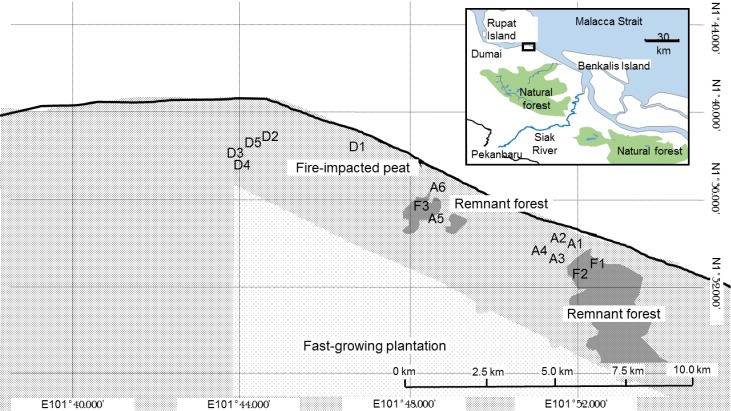
Map of the region where the study was conducted showing the relative location of each transect in the study. The inserted map in the upper right corner shows the position of the Giam Siak Kecil–Bukit Batu Biosphere Reserve in Riau, Sumatra.

We sampled termites and ants in the remnant forests, in the fire-impacted peatlands at sites close to the remnant forests, and at sites distant from the remnant forests. We assumed that arthropods in the remnant forests have the potential to colonize and succeed the fire-induced losses in a given landscape. The remnant forests were surveyed and treated as reference sites (F1–F3). Six sites close to the remnant forest were selected: A1–A4 (adjacent sites) were situated close to F1–F2 (10–100 m) and A5–A6 were located close to F3 (60–1000 m) (Fig 1). In addition, five locations within 10 km from the remnant forests (distant sites, D1–D5) were sampled (Fig 1). For logistical reasons, ants were only surveyed at sites D1, 3, and 4. Time since fire and environmental variables (canopy cover, understory vegetation cover, plant litter depth and dry litter weight) were recorded based on Neoh et al. [14]. The level of ecological degradation in the peats was categorized based on Page et al.’s classification [30] (Table 1).

**Table 1:** Description of the seven studied locations in tropical peats in Sumatra, Indonesia.

Name of location	Geographic location	Level of ecological degradation [30]	Time since fire (month)	Canopy cover (%)	Understory vegetation cover (%)	Plant litter depth (cm)	Dry litter weight (g)
Remnant forests							
F1	01°33.493’ N, 101°51.827’E	Low	Unburned	91	50	5	558
F2	01°33.370’ N, 101°48.662’E	Low	Unburned	85	55	5	533
F3	01°35.889’ N, 101°48.480’E	Low	Unburned	87	61	5	495
Sites close to remnant forests							
A1	01°33.564’ N, 101°51.819’E	Moderate	96	40	5	1	210
A2	01°33.627’ N, 101°51.815’E	Moderate	96	35	6	1	150
A3	01°33.424’ N, 101°51.692’E	High	12	0	7	1	240
A4	01°33.473’ N, 101°51.668’E	High	12	0	8	1	132
A5	01°35.841’ N, 101°48.500’E	Moderate to high	24	0	10	1	221
A6	01°36.444’ N, 101°48.615’E	Moderate to high	12	0	2	1	250
Sites distant from remnant forests							
D1	01°38.382’ N, 101°48.756’E	High	6	0	5	1	205
D2	01°38.556’ N, 101°44.220’E	Moderate	96	5	24	1	268.6
D3	01°37.825’ N, 101°43.988’E	High	6	0	4	1	198
D4	01°37.668’ N, 101°43.949’E	High	6	0	6	1	221
D5	01°38.309’ N, 101°44.204’E	Moderate	96	37.8	11.4	1	250

### Termite and ant sampling

To sample termites, we employed a standardized belt transect (100 × 2 m) as described by Jones and Eggleton [31]. The transect was divided into 20 sections of 5 m intervals. The collector spent 1 h on a section (5 × 2 m each) searching through the potential habitats of termites, such as dead tree branches, tree logs, termite galleries, nests, and soil under logs. Soldier and worker termites were collected and stored in 80% ethanol until they were identified. The termites were sorted to species level based on identification keys of the termite fauna of the Indo-Malayan region [32,33,34,35,36]. To sample ants, pitfall traps were set along 100 m transect of 10 m intervals for 48 h. The pitfall traps were filled with soapy water to prevent the trapped insects from escaping. Ants were collected and stored in 80% ethanol until they were identified. The ants were identified based on descriptions by Hashimoto [37] and Bolton [38]. Termite and ant voucher specimens were deposited at the Department of Entomology, National Chung Hsing University, Taiwan. Termites and ants were assigned to feeding groups [39] and functional groups [40,41], respectively (See S1 and S2 Tables).

### Statistical analysis

We produced sample-based rarefaction curves with 50 randomizations for each sampling plot. Computations were carried out using the program EstimateS Version 8.2 [42]. We compared the species richness and feeding and functional groups in each sampling plot using analysis of variance (SPSS Version 18) at α = 0.05.

The species richness data were subjected to fourth-root transformation prior to analysis of similarities. Pairwise resemblance matrices were visualized using a clustering dendrogram based on the Bray-Curtis percentage similarity index for both termite and ant assemblages at each study site. The clusters were then separated using a similarity profile permutation test (SIMPROF procedure). We constructed one heat map for each taxon surveyed based on habitat type and functional group of the insect. The heat map is a color-coded graphical representation of a matrix that reorders rows and columns according to hierarchical clustering. The matrix illustrates the variation in occurrence as a color gradient from light (low abundance) to dark (high abundance). In addition, a similarity percentage analysis (SIMPER procedure) was conducted to determine the percentage of species present in a surveyed site. The analyses were performed using The PRIMER v7 statistical program (PRIMER-E Ltd, Lutton, UK).

Distances among functional groups and habitat types were obtained using CANOCO 5.0 [43]. The feeding and functional groups within each surveyed site were correlated with environmental traits using canonical correspondence analysis (CCA). A Monte-Carlo test with 999 permutations was conducted to test the significance of the ordination axes. A parametric generalized additive model with Gaussian distribution was used to visualize the association between the Shannon diversity index that reflected species evenness.

## Results

### Species richness

**Fig 2:**
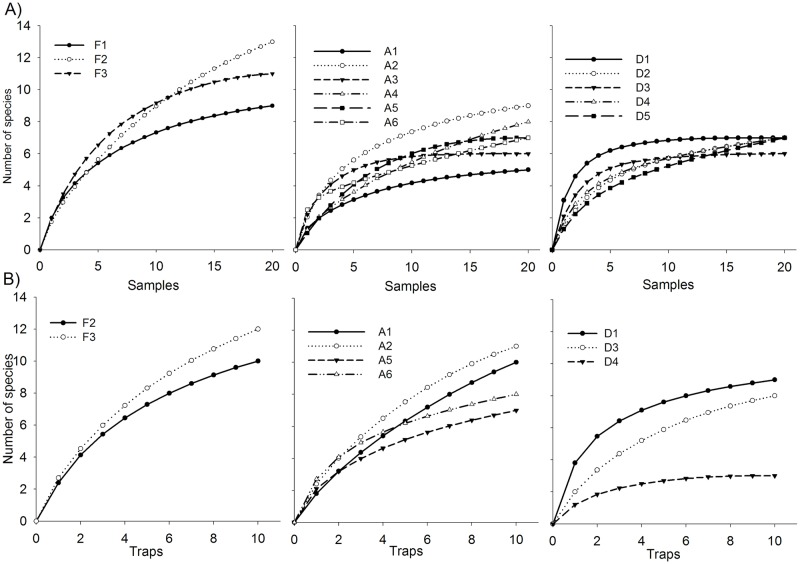
Sample-based rarefaction curves for termite (A) and ant (B) assemblages from standardized 100 m belt transect and pitfall trap samples in fire-impacted peats that were close to and distant from remnant forest compared with assemblages in the remnant forest at Riau, Sumatra.

**Fig 3:**
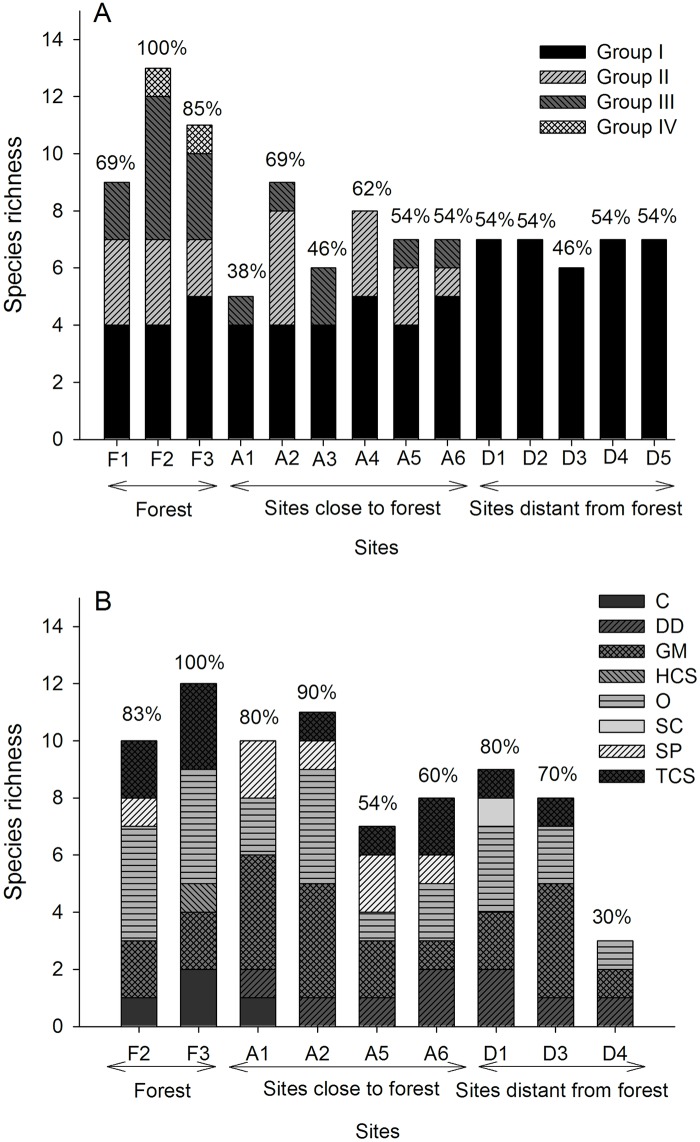
Species richness for termite (A) and ant (B) assemblages from standardized 100 m belt transect and pitfall trap samples in fire-impacted peats that were close to (A1–A6) and distant from remnant forest (D1–D5) compared with assemblages in the remnant forest (F1–F3) at Riau, Sumatra. The termite and ant species encountered were assigned to functional groupings. Abbreviations for termite feeding groups: Group I = feed on dead wood only; Group II = feed on grass, dead wood, and plant litter; Group III = feed on soil with high organic content; Group IV = feed on soil with low organic content. Ant functional groups: C = Cryptic, HCS = Hot-climate Specialists, SP = Specialist Predators, TCS = Tropical-climate Specialists, O = Opportunists, GM = Generalized Myrmicinae, DD = Dominant Dolichoderinae, SC = Subordinate Camponotini. The species richness in each transect is relative to values in the remnant forests (i.e., F2 for termites and F3 for ants).

Twenty-six species of termite from 11 genera were sampled across the 14 sites. In general, the number of termite species in remnant forests was significantly higher than those in fire-impacted peatlands (d.f. = 13, 279; F = 10.435; p < 0.01) in rarefaction curves, but the post-hoc Tukey analyses revealed that the number of species did not differ significantly between sites distant from the forests and sites close to forests (Fig 2A). In all three land use types, Group I species constituted at least 30% of the species richness (Fig 3A). Species richness of Group I at sites distant from the forest was significantly higher than that in the other two land use types (d.f. = 2, 13; F = 38.420; p < 0.05). The species richness of Group II was equal in remnant forests and sites close to the forests, but Group II was absent from sites distant from the forests (d.f. = 2, 13; F = 5.836; p < 0.05). Species richness of Group III in remnant forests was significantly higher than that present in the other two land use types (d.f. = 2, 13; F = 17.365; p < 0.05). Group IV consisted of a single species (d.f. = 2, 13; F = 8.643; p < 0.05), and it was present only in remnant forests.

For ants, 29 species from 8 functional groups were observed. The sample-based rarefaction curves registered 10–12 species in the remnant forests, 8–10 species in fire-impacted peatlands close the remnant forests, and 3–9 species in the distant sites. No significant difference in species richness was detected among the land use types with the exception of site D4 (d.f. = 8, 89; F = 5.856; p < 0.01). At this site, the number of local ant species was at least 70% lower compared to that of remnant forests (Fig 2B). Significantly, more Cryptic (d.f. = 2, 8; F = 7.128; p < 0.05) and Tropical-climate Specialist (d.f. = 2, 8; F = 5.451; p < 0.05) ant species were found in the remnant forests compared to the other land use types. In contrast, the number of species of Dominant Dolichoderinae in remnant forests was significantly lower than that in other land use types (d.f. = 2, 8; F = 7.670; p < 0.05). The species richness of Specialist Predator ants in remnant forests and sites close to the forests was significantly higher than that in distant sites (d.f. = 2, 8; F = 6.644; p < 0.05). The species richness of Generalized Myrmicinae (d.f. = 2, 8; F = 0.169; p > 0.05), Hot-climate Specialists (d.f. = 2, 8; F = 2.333; p > 0.05), Opportunists (d.f. = 2, 8; F = 2.388; p > 0.05), and Subordinate Camponotini (d.f. = 2, 8; F = 1.000; p > 0.05) ants did not differ significantly among all land use types (p > 0.05) (Fig 3B).

### Distribution of functional groups in relation to environmental, ecological degradation, and distance traits

**Fig 4:**
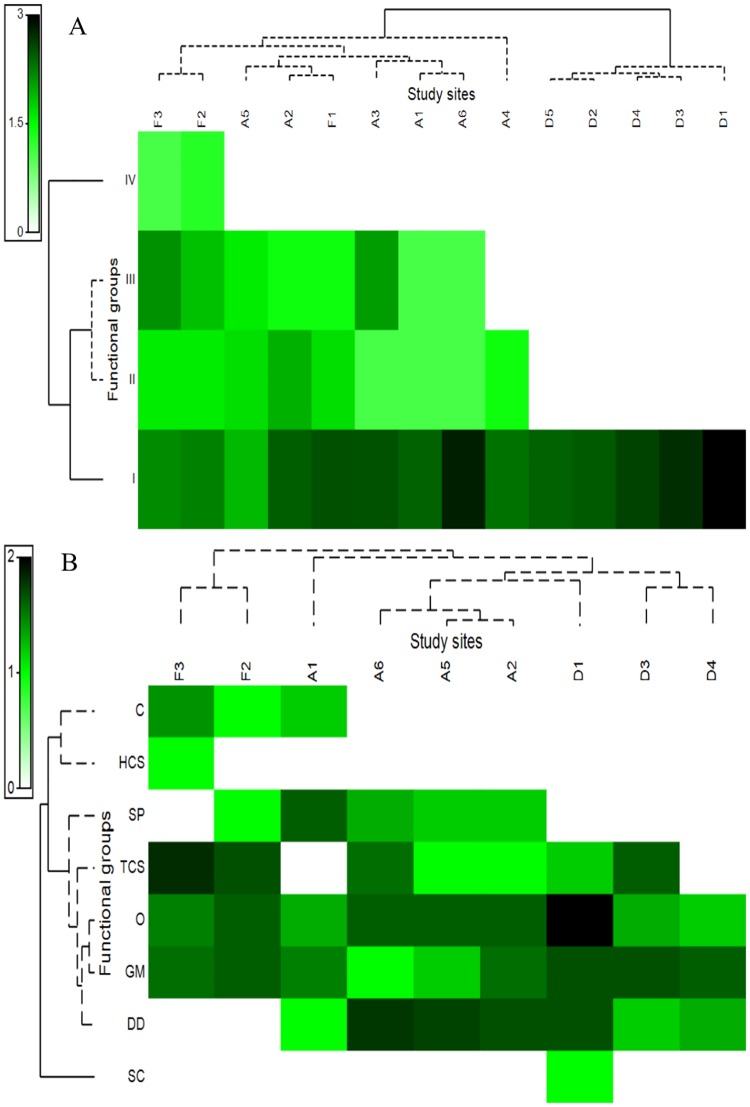
Heat map of species occurrence according to feeding groups of termites (A) and functional groups of ants (B) and habitats. Dark cells represent the highest species occurrence, and light cells represent the lowest. Dendrograms of group-average clustering of functional groups and habitats are based on means of Bray–Curtis distances. Solid lines represent significant differences among groups based on SIMPROF tests (p < 0.05). Abbreviations for feeding and functional groups are as for Fig 3.

**Fig 5:**
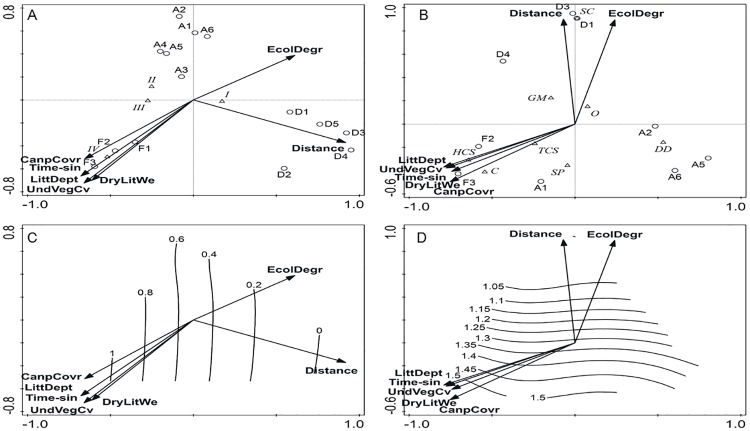
Canonical correspondence analysis (CCA) triplot showing the distribution of termite feeding groups (A) and ant functional groups (B) (triangles) and study sites (white circle) in relation to environmental variables, level of ecological degradation, and distance of site from remnant forest (arrows). The Shannon diversity index values characterizing termite feeding (C) and ant functional groups (D) are shown with isolines fitted with a general additive model. Abbreviations for feeding and functional groups are as for Fig 3. Environmental variables: Distance = distance of study site from the nearest remnant forest, EcolDegr = ecological degradation, LittDept = litter depth, UndVegCv = understory vegetation cover, Time-sin = time since fire, DryLitWe = dry litter weight, CanpCovr = canopy cover.

Functional group composition of both taxa varied with habitat type. For the termites, the sites distant from the forests differed significantly from those in the remnant forest sites and sites close to the forests, which were grouped into two clusters (remnant + close and distant) (Fig 4A). SIMPER analysis revealed that among the functional groups, Groups II and III contributed up to 79.8% of the dissimilarity to separate the two clusters. The CCA determined that 76.8% of the total variation in the data was explained. The distribution of the termite feeding groups in all tested land use types along the two canonical axes was not random (F-ratio = 3.9, P = 0.005, 999 permutations). Litter depth, canopy and understory vegetation cover, and dry litter weight were positively associated with the termite feeding groups, notably Group IV, which was present only in remnant forests (as evidenced from the strong positive association detected for the ‘Time since fire’ explanatory variable). In contrast, the association between Groups II and III and the environmental variables listed above was weak and present in old- and recent-burned fire-impacted peats (Fig 5A). Group I was mainly found in recently burned fire-impacted peats with increased ecological degradation and distance from the remnants forests (Fig 4A). However, it is worth noting that this group was omnipresent, representing 97.5% of the feeding group composition, and showed high abundance in all habitat types based on SIMPER analysis.

For ants, no significant cluster was formed along the land use types based on ant functional group composition. Nevertheless, three main functional groups were found (Fig 4B). The CCA revealed that 93.3% of the variation could be explained, with axis 1 explaining 38.7% and axis 2 accounting for 26.5% of the variation. The distribution of ant functional groups in the analysis along the two canonical axes was not random (F-ratio = 4.7, P = 0.01, 999 permutations). The first group, Cryptic and Hot-climate Specialist ants, was positively associated with litter depth, canopy and understory vegetation cover, and dry litter weight. These ants were exclusively dominant in old growth land uses such as in remnant forests and at a site that was closes to the remnant forest (A1) (Fig 4B). The second group included Specialist Predators, Tropical-climate Specialists, Opportunists, Generalized Myrmicinae and Dominant Dolichoderinae. However, the association between the Specialist Predators, Tropical-climate Specialists, and Generalized Myrmicinae ants and the habitat characteristics mentioned above was weak (Fig 5B). The occurrence of Tropical-climate Specialists, Opportunists, and Generalized Myrmicinae was relatively constant in all habitats regardless of time since fire (Fig 5B). Specialist Predators were consistently found in remnant forests and sites close to remnant forests, but they were very uncommon in sites distant from the remnant forests (Fig 4B). Dominant Dolichoderinae occurred exclusively in fire-impacted peatlands regardless of distance from remnant forests and time since fire, thus they showed no relationship with the environmental variables tested. The last group consisted of Subordinate Camponotini, which were found only in sites distant from remnant forests with characteristics of high ecological degradation.

### Species evenness

In general, the isolines of termite species evenness in the remnant forests and fire-impacted peats located close to the remnant forests corresponded closely with explanatory variables of time since fire and environmental variables. Termites showed clear variability relative to distance and ecological degradation traits, especially between the remnant forests and fire-impacted peats distant from the remnant forests (Fig 5C). For ants, the low species evenness in distant sites corresponds to a function of distance and level of ecological degradation. Ants showed no clear variability relative to environmental variables and time since fire traits, especially between the remnant forests and fire-impacted peats located close to the remnant forests (Fig 5D).

## Discussion

We detected a pronounced difference in functional group composition of termites between sites close to and sites distant from remnant forests. In particular, sites close to remnant forests contained two additional termite feeding groups so that they shared a similar composition structure with remnant forests. In contrast, a high degree of similarity was detected among ant communities in all land uses surveyed compared to remnant forests. The Shannon diversity index for termites and ants decreased with increasing distance from the remnant forests and level of ecological degradation.

### Effect of fire

Despite the loss of termite species number, which was as high as 50% in some fire-impacted peats compared to the remnant forests, the species richness for termites was statistically equal along fire-impacted peatlands regardless of distance from the remnant forests. However, there was a pronounced difference in feeding group composition of termite communities with distance from the remnant forests. In particular, only the termite family Rhinotermitidae was present in the distant fire-impacted peatlands. Thus, the influence of peat fire on the termite community is obvious. Our previous study showed that termites belonging to the family Termitidae were among the most affected by fire in tropical degraded peatland [44]. The high temperature of peat fires and the smoldering effect could have driven the collapse of these exclusively nest-building termites, whereas rhinotermitids that adaptively nest in tree barks could have survived the effects of fire. Thus, the presence of termitids (Groups II and III) in fire-impacted peatlands located close to remnant forests most likely indicates recolonization.

Our analysis of the ant data suggests that no land use type was significantly different in term of species richness and the Bray-Curtis index based on functional group occurrence. This indicates that peat fires have a negligible effect on ant species richness. The insensitivity of ant species richness to fires agrees somewhat with a previous study conducted in the Brazilian Cerrado wetland [45]. It was reasoned that any loss of ant species in a given site can be offset by gains of other generalist ants, so the effect on community composition in association with the ants’ functional roles in an ecosystem is pronounced [45]. However, the effects of fire are very habitat specific. The present results are somewhat contradictory, as no significant difference in ant functional group composition between the burned sites and remnant forests was detected.

Fire could act as an ecological filter to eliminate the ant species that are most vulnerable to fires and post-fire effects in association with changes in vegetation structure and composition, canopy openness, and soil characteristics [19,46,47,48]. Few of our ant functional groups, including Tropical-climate Specialists, Opportunists, Generalized Myrmicinae, and Dominant Dolichoderinae, appeared to be diagnostic functional groups in fire-impacted peatlands. However, the Cryptic ants and Specialized Predator ants were absent from the burned sites. Although the ant functional group composition remained the same across all types of land use, the overall abundance of ants in distant sites increased due to the dominance of aggressive or exotic species. These findings corroborate the Shannon diversity index results, which showed that the evenness of functional diversity decreased with increased distance from remnant forests. In particular, *Anoplolepis gracilipes* Smith, *Iridomyrmex* sp., *Paratrechina* sp., and *Pheidole* sp. were recorded in high frequency in the distant fire-impacted peatlands (S2 Table). Species from these genera are known to be behaviorally and ecologically dominant, with high abundance and resource monopolization, in highly disturbed landscapes. These ants have been recognized as dominant species in many ant communities in disturbed habitats worldwide, where they suppress invertebrate biodiversity via competitive exclusion [49,50,51,52,53].

### Long-term impact of fire

A simplified termite community structure and dominance of Group I termites were observed in distant fire-impacted peatlands. This was especially true at sites D2 and D5, where the reduced termite composition structure was still readily recognizable even though the fire took place in 2007. In the semi-arid mallee region in Southeastern Australia, habitat heterogeneity played a more pronounced role in shaping the termite assemblage compared to time since the fire [18]. In our study, the vegetation structure became complex at sites D2 and D5 over time, but the complexity of vegetation structure did not favor termite colonization. For ants, we only sampled at distant sites that were recently burned in the present study, so our ant results are not conclusive. However, poor dispersal ability [54] and unfavorable conditions in the oil palm plantation [50] at the study site may have created an effective dispersal barrier for forest ant colonization in the distant fire-impacted peats, as has been shown for butterflies [55]. Floren and Linsenmair [56], who investigated the effects of logging activity on arboreal ants in a tropical rain forest, reported that the loss of ant diversity and changes in community structure in disturbed land use areas were obvious even in a 40 year old regenerated forest. In an Amazonian forest, Silveira et al. [46] found that the differences in ant species communities between unburned and burned forests remained apparent 10 years after fire disturbance. These previous studies reinforce the suggestion that the impact of fire on termite and ant taxa that results in local extinction of forest species and the dominance of invasive and tramp ants and wood-feeding termites in the distant peat sites could be a long-term effect.

### Recolonization

In general, soil-feeding termites are particularly sensitive to fragmentation; they showed a 20% reduction of total species richness compared to the scenario in Amazonian continuous forests [57]. Similarly, the ant assemblage in a tropical rain forest was significantly reduced and community composition was simplified due to fragmentation [58,59]. Ants with specialized habits tend to be more sensitive to forest fragmentation compared to opportunist and generalist ant species [60]. In the present study, the peat swamp forest sites surveyed were smaller than 5 ha. We did not sample in continuous peat swamp forests, so it is not possible to know if the species assemblage was affected by fragmentation. However, the fragmented forests support disturbance-sensitive species of both taxa, such as soil-feeding termites (*Subulitermes* group), and Specialist Predators and Generalized Myrmicinae ants that reportedly are strongly associated with forest systems [61]. Given the rich biodiversity of remnant peat swamp forests, these remnants can act as sources for recolonization of adjacent areas.

As a rule, recovery of insect biodiversity depends on habitat quality following fire events and insect dispersal ability [62]. Avitabile et al. [18] showed that the effect of vegetation change after a fire on the overall termite species and community was limited as long as dead wood as termite food continued to be available. An experimental habitat perturbation demonstrated that dead wood left on the ground not only facilitates the recovery of species richness and abundance but that it also enriches the composition of the termite community [63]. In the present study, the effect of habitat heterogeneity on sites both distant and adjacent to remnant forests was expected to be subtle because the in situ above-ground vegetation and canopy openness in both fire-impacted peatlands were homogenous. Although ant community composition was not statistically different among land use types in the present study, there is good evidence to support the hypothesis that the presence of forest-adapted functional groups (i.e., Cryptic and Specialized Predator ants) in sites close to remnant forests would reflect the potential of forests to act as a biodiversity reservoir to facilitate the recolonization process. Research conducted in a Southeast Asian tropical rain forest showed that ant species richness in oil palm plantation was higher when the plantation was close to forest [28]. In programs designed to rehabilitate Australian bauxite mines, plant species richness and diversity, time since rehabilitation, percentage of plant cover and litter cover, and availability of logs affected recolonization by opportunist ants, but these factors failed to promote the return of forest ants [64]. In addition, the high evenness of functional diversity in fire-impacted sites close to remnant forests indicates that forests could act as a natural buffer and limit the dominance of certain ant species while enhancing species diversity via recolonization of forest-adapted species. The dominance of tramp ants probably is buffered by limited resource monopolization and foraging area and increased interspecific competition. In our study we did not address the question of how far away recolonization could occur. However, the maximum distance of the recolonization effect may be 1 km from the remnant forest, as this is the farthest termite dispersal distance recorded to date [65]. Working with the invertebrate community associated with the plant *Sporadanthus ferrugineus* (Restionaceae) in peat bogs, Watts and Didham [66] reported that the total number of taxa recorded in potted plants located 800 m from an intact peat bog decreased to 38% of the number present in the peat bog compared to 62% on potted plants at a distance of 30 m.

Several studies have shown how important natural or semi-natural areas are for providing a vital ecosystem service to nearby disturbed lands. Working with bees in canola fields in southern Alberta, Canada, Morandin et al. [67] demonstrated that fields with high pastureland cover contained significantly more native bee species, which might have contributed to the high pollination rate compared to that in fields with low plant cover. In a Costa Rican coffee agroecosystem, tropical forest remnants significantly enhanced species richness of bees and the pollination rate in sites located close to the fragments [68]. Similar observations were made for butterflies in tropical rain forest fragments [28,69]. In the present study, recolonization by ant and termite taxa may have facilitated post-fire ecosystem restoration. For example, Donovan et al. [70] showed that a soil-feeding termite (Group III) and increased soil pH, organic carbon, and water content significantly modified soil composition to favor plant growth. Termite nests create spatial structure for plant survival and growth and increase functional diversity of woody plants [71,72,73,74] and also provide refugia for multiple taxa [75,76]. In addition to the roles of species within functional groups of Specialist Predators and Cryptic ants, their effects on a variety of ecological processes have been widely recognized [9]. The introduction of forest ant species from remnant forests indirectly alters the ant dominance structure in fire-impacted peats, which favors ecosystem processes such as species coexistence in a given site [77].

## Conclusion

Drainage and peat fires can have detrimental effects on the hydrophysical properties of peat. The combined effects of both disturbances result in increased soil bulk density, water retention, and loss of moisture content [78,79]. The heat produced by peat fires potentially causes massive loss of peat mass and thus creates nutrient-poor peat [80]. Such changes make the plant regeneration process impossible and the cultivation of many agricultural crops difficult. The successional processes of termites and ants in the tropical peat swamp forest ecosystem following disturbances may have important implications for ecosystem function restoration and the ecological dynamics of species. In the present study, we were unable to fully address the generality of the trends observed in the tropical peat swamps in the Indo-Malayan region, as factors such as the effect of spatial autocorrelation in the distant sites might affect interpretation of results, and variation in fire intensity was not included as an explanatory variable. Nevertheless, we provide evidence supporting the value of conserving remnant peat forests in Southeast Asia, where tropical peats are continuously being threatened and exploited for more oil palm and timber plantations [81].

## Supporting information

S1 Table Abundance of termite assemblages in remnant forests, sites close to remnant forests, and sites distant from remnant forests. The termites were categorized based on feeding groups: Group I: termites that feed only on wood (mainly Rhinotermitidae); Group II: termites that have a wide range of food types, including wood, plant litter, and microepiphytes; Group III: termites that feed on soil with high organic content or highly decayed soil-like wood; Group IV: termites that feed on soil with low organic content [38].
(PDF)

S2 Table Abundance of ant assemblages in remnant forests, sites close to remnant forests, and sites distant from remnant forests. Ants were categorized based on functional groups: Cryptic (C): abundant and diverse in forests, nest underground or in dead plant materials; Dominant Dolichoderinae (DD): numerically and behaviorally dominant group in open environments; Generalized Myrmicinae (GM): widespread group, armed with chemical defenses and often show resource monopolization; Hot-climate Specialists (HCS): highly adaptable to extreme heat and distribution limited to arid regions; Opportunists (O): widespread group, especially when ant diversity is low, and less competitive and unspecialized in ecological function; Subordinate Camponotini (SC): generally present in areas with high diversity of the ant community and often show apparent niche separation with DD; Specialist Predators (SP): specialization on certain arthropod prey; Tropical-climate Specialists (TCS): Hot-specialized ant group with distribution limited to the tropics [39,40].
(PDF)

## References

[1] Miettinen J, Shi C, Liew SC. Two decades of destruction in Southeast Asia's peat swamp forests. Front Ecol Environ. 2011; 10: 124–128.

[2] Yulianti N, Hayasaka H, Usup A. Recent forest and peat fire trends in Indonesia: The latest decade by MODIS hotspot data. Global Environ Res. 2012; 16: 105–116.

[3] Relys V, Koponen S, Dapkus D. Annual differences and species turnover in peat bog spider communities. J Arachnol. 2002; 30: 416–424.

[4] Carrera N, Barreal ME, Gallego PP, Briones MJI. Soil invertebrates control peatland C fluxes in response to warming. Funct Ecol. 2009; 23: 637–648.

[5] Drinan TJ, Foster GN, Nelson BH, O'Halloran J, Harrison SSC. Macroinvertebrate assemblages of peatland lakes: Assessment of conservation value with respect to anthropogenic land-cover change. Biol Conserv. 2013; 158: 175–187.

[6] Coulson JC, Butterfield JEL. The invertebrate communities of peat and upland grasslands in the north of England and some conservation implications. Biol Conserv. 1985; 34: 197–225.

[7] Wiecek M, Martin P, Lipinski A. Water mites as potential long-term bioindicators in formerly drained and rewetted raised bogs. Ecol Indic. 2013; 34: 332–335.

[8] Scott AG, Oxford GS, Selden PA. Epigeic spiders as ecological indicators of conservation value for peat. Biol Conserv. 2006; 127: 420–428.

[9] Del Toro I, Ribbons RR, Pelini SL. The little things that run the world revisited: A review of ant-mediated ecosystem services and disservices (Hymenoptera: Formicidae). Myrmecol. News. 2012; 17: 133–146.

[10] Bignell DE, Eggleton P. Termites in Ecosystems In: Abe T, Bignell DE, Higashi M, editors. Termite: Evolution, Sociality, Symbioses, Ecology. Dordrecht: Kluwer Academic Publishers; 2000 pp 363–388

[11] Hyodo F, Matsumoto T, Takematsu Y, Itioka T. Dependence of diverse consumers on detritus in a tropical rain forest food web as revealed by radiocarbon analysis. Funct Ecol. 2014; 29: 423–429.

[12] Hyodo F, Takematsu Y, Matsumoto T, Inui Y, Itioka T. Feeding habits of Hymenoptera and Isoptera in a tropical rain forest as revealed by nitrogen and carbon isotope ratios. Insect Soc. 2011; 58: 417–426.

[13] Jones DT, Susilo FX, Bignell DE, Hardiwinoto S, Gillison AN, Eggleton P. Termite assemblage collapse along a land-use intensification gradient in lowland central Sumatra, Indonesia. J Appl Ecol. 2003; 40: 380–391.

[14] Neoh K-B, Bong L-J, My NT, Vuong NT, Quoc HN, Itoh M, et al Termite diversity and complexity in Vietnamese agroecosystems along a gradient of increasing disturbance. J Insect Conserv. 2015; 19: 1129–1139.

[15] Hoffmann BD, Andersen AN. Responses of ants to disturbance in Australia, with particular reference to functional groups. Aust Ecol. 2003; 28: 444–464.

[16] Neoh K-B, Bong L-J, Muhammad A, Itoh M, Kozan O, Takematsu Y, et al Understanding the impact of fire on termites in degraded tropical peatlands and the mechanisms for their ecological success: current knowledge and research needs. Ecol Res. 2015; 30: 759–769.

[17] Davies AB, Eggleton P, Van Rensburg BJ, Parr CL. The pyrodiversity-biodiversity hypothesis: A test with savanna termite assemblages. J Appl Ecol. 2012; 49: 422–430.

[18] Avitabile SC, Nimmo DG, Bennett AF, Clarke MF. Termites are resistant to the effects of fire at multiple spatial scales. PLoS ONE. 2015; 10: e0140114 10.1371/journal.pone.0140114 26571383 PMC4646461

[19] Vasconcelos HL, Leite MF, Vilhena JMS, Lima AP, Magnusson WE. Ant diversity in an Amazonian savanna: Relationship with vegetation structure, disturbance by fire, and dominant ants. Aust Ecol. 2008; 33: 221–231.

[20] Vasconcelos HL, Pacheco R, Silva RC, Vasconcelos PB, Lopes CT, Costa AN, et al Dynamics of the leaf-litter arthropod fauna following fire in a neotropical woodland savanna. PLoS ONE 2009; 4: e7762 10.1371/journal.pone.0007762 19898619 PMC2768909

[21] Moranz RA, Debinski DM, Winkler L, Trager J, McGranahan DA, Engle DM, et al Effects of grassland management practices on ant functional groups in central North America. J Insect Conserv. 2013; 17: 699–713.

[22] Posa MRC, Wijedasa LS, Corlett RT. Biodiversity and conservation of tropical peat swamp forests. BioScience. 2011; 61: 49–57.

[23] Yule CM. Loss of biodiversity and ecosystem functioning in Indo-Malayan peat swamp forests. Biodivers Conserv. 2010; 19: 393–409.

[24] Hooper ER, Legendre P, Condit R. Factors affecting community composition of forest regeneration in deforested, abandoned land in Panama. Ecology. 2004; 85: 3313–3326.

[25] Anand MO, Krishnaswamy J, Kumar A, Bali A. Sustaining biodiversity conservation in human-modified landscapes in the Western Ghats: Remnant forests matter. Biol Conserv. 2010; 143: 2363–2374.

[26] Gray CL, Simmons BI, Fayle TM, Mann DJ, Slade EM. Are riparian forest reserves sources of invertebrate biodiversity spillover and associated ecosystem functions in oil palm landscapes? Biol Conserv. 2016; 194: 176–183.

[27] Lucey JM, Hill JK. Spillover of Insects from Rain Forest into Adjacent Oil Palm Plantations. Biotropica. 2012; 44: 368–377.

[28] Lucey JM, Tawatao N, Senior MJM, Chey VK, Benedick S, Hamer KC, et al Tropical forest fragments contribute to species richness in adjacent oil palm plantations. Biol Conserv. 2014; 169: 268–276.

[29] Livingston G, Jha S, Vega A, Gilbert L. Conservation value and permeability of neotropical oil palm landscapes for orchid bees. PLoS ONE. 2013 8: e78523 10.1371/journal.pone.0078523 24147137 PMC3798381

[30] Page S, Hoscilo A, Langner A, Tansey K, Siegert F, Limin S, et al Tropical peatland fires in Southeast Asia Tropical Fire Ecology: Springer Berlin Heidelberg; 2009 pp. 263–287.

[31] Jones DT, Eggleton P. Sampling termite assemblages in tropical forests: testing a rapid biodiversity assessment protocol. J Appl Ecol. 2000; 37: 191–203.

[32] Thapa RS. Termites of Sabah. Sandakan: Sabah Forestry Department 1997.

[33] Gathorne-Hardy FJ. The termites of Sundaland: A taxonomic review. Sarawak Museum Journal; 2004 pp. 88–133.

[34] Tho YP. Termites of peninsular Malaysia. Kuala Lumpur: Forest Research Institute of Malaysia 1992.

[35] Jones DT, Prasetyo AH. A survey of the termites (Insecta: Isoptera) of Tabalong District, South Kalimantan, Indonesia. Raffles Bull Zool. 2002; 50: 117–128.

[36] Jones DT, Brendell MJD. The termite (Insecta: Isoptera) fauna of Pasoh Forest Reserve, Malaysia. Raffles Bull Zool. 1998; 46: 79–91.

[37] Hashimoto Y. Identification guide to the ant genera of Borneo Inventory and collection. UMS-BBEC Press, Kota Kinabalu 2003 pp. 95–160.

[38] Bolton B. Identification guide to the ant genera of the world. Cambridge: Harvard University Press 1994

[39] Donovan SE, Eggleton P, Bignell DE. Gut content analysis and a new feeding group classification of termites. Ecol Entomol. 2001; 26: 356–366.

[40] Andersen AN. A global ecology of rainforest ants: Functional groups in relation to environmental stress and disturbance In: Agosti D, Majer J, Alonso L, Schultz T, editors. Ants: standard methods for measuring and monitoring biodiversity, biological. Washington: Smithsonian Institution Press; 2000 pp. 25–34.

[41] Brown WL Jr. Diversity of ants In: Agosti D, Majer J, Alonso L, Schultz T, editors. Ants: Standard methods for measuring and monitoring biodiversity. Washington: Smithsonian Institute Press; 2000 pp. 45–79.

[42] Colwell RK. EstimateS: Statistical estimation of species richness and shared species from samples Version 82 User's Guide and application. 2005. http://purloclcorg/estimates.

[43] ter Braak C, Smilauer P. Canoco reference manual and user’s guide: Software for ordination (version 5.0). Ithaca, NY, USA Microcomputer Power; 2012.

[44] Neoh K-B, Bong L-J, Muhammad A, Itoh M, Kozan O, Takematsu Y, et al The impact of tropical peat fire on termite assemblage in Sumatra, Indonesia: Reduced complexity of community structure and survival strategies. Environ Entomol. 2016; 45: 1170–1177. 10.1093/ee/nvw116 27550162

[45] Costa-Milanez CB, Ribeiro FF, Castro PTA, Majer JD, Ribeiro SP. Effect of fire on ant assemblages in Brazilian cerrado in areas containing vereda wetlands. Sociobiology. 2015; 62: 494–505.

[46] Silveira JM, Barlow J, Andrade RB, Louzada J, Mestre LA, Lacau S. et al The responses of leaf litter ant communities to wildfires in the Brazilian Amazon: A multi-region assessment. Biodivers Conserv. 2013; 22: 513–529.

[47] Parr CL, Robertson HG, Biggs HC, Chown SL. Response of African savanna ants to long-term fire regimes. J Appl Ecol. 2004; 41: 630–642.

[48] Arnan X, Rodrigo A, Retana J. Post-fire recovery of Mediterranean ground ant communities follows vegetation and dryness gradients. J Biogeogr. 2006; 33: 1246–1258.

[49] Hoffmann BD, Andersen AN, Hill GJ. Impact of an introduced ant on native rain forest invertebrates: *Pheidole megacephala* in monsoonal Australia. Oecologia. 1999; 120: 595–604. 28308311 10.1007/PL00008824

[50] Brühl CA, Eltz T. Fuelling the biodiversity crisis: species loss of ground-dwelling forest ants in oil palm plantations in Sabah, Malaysia (Borneo). Biodivers Conserv. 2010; 19: 519–529.

[51] Gibb H, Hochuli DF. Colonisation by a dominant ant facilitated by anthropogenic disturbance: Effects on ant assemblage composition, biomass and resource use. Oikos. 2003; 103: 469–478.

[52] Wetterer JK, Miller S, Wheeler D, Olson C, Polhemus D, Pitts M, et al Ecological dominance by *Paratrechina longicornis* (Hymenoptera: Formicidae), an invasive tramp ant, in biosphere 2. Fla Entomol. 1999 pp. 381–388.

[53] Bos MM, Tylianakis JM, Steffan-Dewenter I, Tscharntke T. The invasive yellow crazy ant and the decline of forest ant diversity in Indonesian cacao agroforests. Biol Invasions. 2008; 10: 1399–1409.

[54] Bickel TO, Brühl CA, Gadau JR, Hölldobler B, Linsenmair KE. Influence of habitat fragmentation on the genetic variability in leaf litter ant populations in tropical rainforests of Sabah, Borneo. Biodivers Conserv. 2006; 15: 157–175.

[55] Scriven SA, Beale CM, Benedick S, Hill JK. Barriers to dispersal of rain forest butterflies in tropical agricultural landscapes. Biotropica. 2016;

[56] Floren A, Linsenmair KE. The Importance of primary tropical rain forest for species diversity: An investigation using arboreal ants as an example. Ecosystems. 2005; 8: 559–567.

[57] de Souza OFF, Brown VK. Effects of habitat fragmentation on Amazonian termite communities. J Trop Ecol. 1994; 10: 197–206.

[58] Brühl CA, Eltz T, Linsenmair KE. Size does matter–effects of tropical rainforest fragmentation on the leaf litter ant community in Sabah, Malaysia. Biodivers Conserv. 2003; 12: 1371–1389.

[59] Tawatao N, Lucey JM, Senior M, Benedick S, Vun Khen C, Hill JK, et al Biodiversity of leaf-litter ants in fragmented tropical rainforests of Borneo: The value of publically and privately managed forest fragments. Biodivers Conserv. 2014; 23: 3113–3126.

[60] Leal IR, Filgueiras BKC, Gomes JP, Iannuzzi L, Andersen AN. Effects of habitat fragmentation on ant richness and functional composition in Brazilian Atlantic forest. Biodivers Conserv. 2012; 21: 1687–1701.

[61] Luke SH, Fayle TM, Eggleton P, Turner EC, Davies RG. Functional structure of ant and termite assemblages in old growth forest, logged forest and oil palm plantation in Malaysian Borneo. Biodivers Conserv. 2014; 23: 2817–2832.

[62] Swengel AB. A literature review of insect responses to fire, compared to other conservation managements of open habitat. Biodivers. Conserv. 2001; 10: 1141–1169.

[63] Davies RG, Eggleton P, Dibog L, Lawton JH, Bignell DE, Brauman A, et al Successional response of a tropical forest termite assemblage to experimental habitat perturbation. J Appl Ecol. 1999; 36: 946–962.

[64] Majer J, Day J, Kabay E, Perriman W. Recolonization by ants in bauxite mines rehabilitated by a number of different methods. J Appl Ecol. 1984; 21: 355–375.

[65] Messenger MT, Mullins AJ. New flights distance recorded for *Coptotermes formosanus* (Isoptera: Rhinotermitidae). Fla Entomol. 2005; 88: 99–100.

[66] Watts CH, Didham RK. Influences of habitat isolation on invertebrate colonization of *Sporadanthus ferrugineus* in a mined peat bog. Restor Ecol. 2006; 14: 412–419.

[67] Morandin LA, Winston ML, Abbott VA, Franklin MT. Can pastureland increase wild bee abundance in agriculturally intense areas? Basic Appl Ecol. 2007; 8: 117–124.

[68] Ricketts TH. Tropical forest fragments enhance pollinator activity in nearby coffee crops. Conserv Biol. 2004; 18: 1262–1271.

[69] Benedick S, Hill J, Mustaffa N, Chey V, Maryati M, Searle J, et al Impacts of rain forest fragmentation on butterflies in northern Borneo: species richness, turnover and the value of small fragments. J Appl Ecol. 2006; 43: 967–977.

[70] Donovan SE, Eggleton P, Dubbin WE, Batchelder M, Dibog L. The effect of a soil-feeding termite, *Cubitermes fungifaber* (Isoptera: Termitidae) on soil properties: termites may be an important source of soil microhabitat heterogeneity in tropical forests. Pedobiologia. 2001; 45: 1–11.

[71] Joseph G, Seymour C, Cumming G, Cumming DM, Mahlangu Z. Termite mounds increase functional diversity of woody plants in African Savannas. Ecosystems. 2014; 17: 808–819.

[72] Beaudrot L, Du Y, Rahman Kassim A, Rejmánek M, Harrison RD. Do epigeal termite mounds increase the diversity of plant habitats in a tropical rain forest in Peninsular Malaysia? PLoS ONE. 2011; 6(5): e19777 10.1371/journal.pone.0019777 21625558 PMC3098262

[73] Fox-Dobbs K, Doak DF, Brody AK, Palmer TM. Termites create spatial structure and govern ecosystem function by affecting N^2^ fixation in an East African savanna. Ecology. 2010; 91: 1296–1307. 20503863 10.1890/09-0653.1

[74] Moe SR, Mobaek R, Narmo AK. Mound building termites contribute to savanna vegetation heterogeneity. Plant Ecol. 2009; 202: 31–40.

[75] Joseph GS, Cumming GS, Cumming DHM, Mahlangu Z, Altwegg R, Seymour CL. Large termitaria act as refugia for tall trees, deadwood and cavity-using birds in a miombo woodland. Landscape Ecol. 2011; 26: 439–448.

[76] Joseph GS, Seymour CL, Cumming GS, Mahlangu Z, Cumming DHM. Escaping the flames: Large termitaria as refugia from fire in miombo woodland. Landscape Ecol. 2013; 28: 1505–1516.

[77] Hillebrand H, Bennett DM, Cadotte MW. Consequences of dominance: A review of evenness effects on local and regional ecosystem processes. Ecology. 2008; 89: 1510–1520. 18589516 10.1890/07-1053.1

[78] Sherwood JH, Kettridge N, Thompson DK, Morris PJ, Silins U, Waddington JM. Effect of drainage and wildfire on peat hydrophysical properties. Hydrol Process. 2013; 27: 1866–1874.

[79] Thompson DK, Waddington JM. Peat properties and water retention in boreal forested peatlands subject to wildfire. Water Resour Res. 2013; 49: 3651–3658.

[80] Rein G, Cleaver N, Ashton C, Pironi P, Torero JL. The severity of smouldering peat fires and damage to the forest soil. Catena. 2008; 74: 304–309.

[81] Hooijer A, Page S, Canadell JG, Silvius M, Kwadijk J, Wösten H, et al Current and future CO_2_ emissions from drained peatlands in Southeast Asia. Biogeosciences. 2010; 7: 1505–1514.

