# Supplementary material for: The effect of remnant forest on insect successional response in tropical fire-impacted peatland: A bi-taxa comparison
Source: PLoS One. 2017 Mar 23;12(3):e0174388. doi: 10.1371/journal.pone.0174388 (PMC5363919; doi:10.1371/journal.pone.0174388)
Supplement: S1 Table — The termites were categorized based on feeding groups: Group I: termites that feed only on wood (mainly Rhinotermitidae); Group II: termites that have a wide range of food types, including wood, plant litter, and microepiphytes; Group III: termites that feed on soil with high organic content or highly decayed soil-like wood; Group IV: termites that feed on soil with low organic content [38]. (PDF) [file pone.0174388.s001.pdf]

S1 Table. Abundance of termite assemblages in remnant forests, sites close to remnant forests, and sites distant from remnant forests. The termites were categorized based on feeding groups: Group I: termites that feed only on wood (mainly Rhinotermitidae); Group II: termites that have a wide range of food types, including wood, plant litter, and microepiphytes; Group III: termites that feed on soil with high organic content or highly decayed soil-like wood; Group IV: termites that feed on soil with low organic content [38].

Termite species	Remnant forests			Sites distant from remnant forests					Sites close to remnant forests					
	F1	F2	F3	D1	D2	D3	D4	D5	A1	A2	A3	A4	A5	A6
Group I														
Coptotermes curvignathus	0	1	0	0	0	0	0	0	0	0	0	0	2	0
Coptotermes gestroi	0	2	2	7	4	9	7	0	0	0	0	2	0	2
Coptotermes kalshoveni	4	0	0	9	1	8	8	1	4	4	6	4	2	11
Coptotermes sp. 1	0	0	0	3	1	0	0	0	0	0	0	0	0	1
Coptotermes sp. 2	0	0	0	0	0	0	0	1	0	0	0	0	0	0
Coptotermes sp. 3	0	0	0	0	0	0	1	1	0	0	0	0	0	0
Parrhinotermes aequalis	4	0	5	13	6	6	4	6	0	0	0	0	0	0
Parrhinotermes pygmaues	10	2	2	11	6	2	1	2	2	2	5	1	0	0
Schedorhinotermes malaccensis	0	0	2	4	2	6	2	5	4	11	3	2	2	18
Schedorhinotermes medioobscurus	13	14	6	15	8	11	12	10	16	10	16	13	4	16
Group II														
Amitermes dentatus	0	0	0	0	0	0	0	0	0	0	0	1	0	0
Microcerotermes havilandi	0	1	0	0	0	0	0	0	0	1	0	0	3	0
Bulbitermes borneensis	0	0	3	0	0	0	0	0	0	2	0	0	3	0
Bulbitermes sarawakiensis	1	3	0	0	0	0	0	0	0	0	0	1	0	0
Nasutitermes havilandi	0	0	0	0	0	0	0	0	0	7	0	0	0	0

Nasutitermes neoparvus	0	1	0	0	0	0	0	0	0	0	0	0	0	0
Nasutitermes proatripennis	3	0	2	0	0	0	0	0	0	0	0	0	0	1
Nasutitermes regularis	2	0	0	0	0	0	0	0	0	1	0	0	0	0
Nasutitermes sp.	0	0	0	0	0	0	0	0	0	0	0	1	0	0
Group III														
Pericapritermes nitobei	0	3	0	0	0	0	0	0	1	3	10	0	0	1
Pericapritermes semarangi	1	1	2	0	0	0	0	0	0	0	4	0	0	0
Pericapritermes speciosus	0	1	5	0	0	0	0	0	0	0	0	0	0	0
Pericapritermes latignathus	0	0	0	0	0	0	0	0	0	0	0	0	5	0
Procapritermes sp.	2	3	9	0	0	0	0	0	0	0	0	0	0	0
Termes rostratus	0	1	0	0	0	0	0	0	0	0	0	0	0	0
Group IV														
Subulitermes group	0	2	1	0	0	0	0	0	0	0	0	0	0	0
