# Supplementary material for: The effect of remnant forest on insect successional response in tropical fire-impacted peatland: A bi-taxa comparison
Source: PLoS One. 2017 Mar 23;12(3):e0174388. doi: 10.1371/journal.pone.0174388 (PMC5363919; doi:10.1371/journal.pone.0174388)
Supplement: S2 Table — Ants were categorized based on functional groups: Cryptic (C): abundant and diverse in forests, nest underground or in dead plant materials; Dominant Dolichoderinae (DD): numerically and behaviorally dominant group in open environments; Generalized Myrmicinae (GM): widespread group, armed with chemical defenses and often show resource monopolization; Hot-climate Specialists (HCS): highly adaptable to extreme heat and distribution limited to arid regions; Opportunists (O): widespread group, especially when ant diversity is low, and less competitive and unspecialized in ecological function; Subordinate Camponotini (SC): generally present in areas with high diversity of the ant community and often show apparent niche separation with DD; Specialist Predators (SP): specialization on certain arthropod prey; Tropical-climate Specialists (TCS): Hot-specialized ant group with distribution limited to the tropics [39,40]. (PDF) [file pone.0174388.s002.pdf]

S2 Table. Abundance of ant assemblages in remnant forests, sites close to remnant forests, and sites distant from remnant forests. Ants were categorized based on functional groups: Cryptic (C): abundant and diverse in forests, nest underground or in dead plant materials; Dominant Dolichoderinae (DD): numerically and behaviorally dominant group in open environments; Generalized Myrmicinae (GM): widespread group, armed with chemical defenses and often show resource monopolization; Hot-climate Specialists (HCS): highly adaptable to extreme heat and distribution limited to arid regions; Opportunists (O): widespread group, especially when ant diversity is low, and less competitive and unspecialized in ecological function; Subordinate Camponotini (SC): generally present in areas with high diversity of the ant community and often show apparent niche separation with DD; Specialist Predators (SP): specialization on certain arthropod prey; Tropical-climate Specialists (TCS): Hot-specialized ant group with distribution limited to the tropics [39,40].

Ant species	Remnant forests		Sites distant from remnant forests			Sites close to remnant forests			
	F2	F3	D3	D4	D1	A1	A2	A5	A6
Cryptic species (C)									
Pheigeleton sp. 1	500	5	0	0	0	21	0	0	0
Pheigeleton sp. 2	0	4	0	0	0	0	0	0	0
Dominant Dolichoderinae (DD)									
Iridomyrmex anceps	0	0	3	2	97	3	76	36	43
Iridomyrmex sp. 2	0	0	0	0	26	0	0	0	2
Generalized Myrmicinae (GM)									
Pheidole sp. 1	0	4	8	0	5	0	0	0	0
Pheidole sp. 2	0	0	9	23	29	3	11	1	7
Pheidole sp. 3	0	0	1	0	0	0	1	0	0
Pheidole sp. 4	32	8	0	0	0	6	8	7	0
Crematogaster sp. 1	1	0	0	0	0	0	0	0	0

Monomorium sp. 1	0	0	2	0	0	1	4	0	0
Monomorium sp. 2	0	0	0	0	0	5	0	0	0
Hot-climate Specialists (HCS)									
Meranoplus sp. 1	0	1	0	0	0	0	0	0	0
Opportunists (O)									
Paratrechina sp. 1	3	1	5	0	274	35	9	0	14
Nylanderia sp. 1	2	4	0	0	24	1	5	6	16
Diacamma sp. 1	2	0	0	3	0	0	0	0	0
Tetramorium sp. 1	1	0	0	0	0	0	0	0	0
Tapinoma sp. 1	0	1	0	0	1	0	0	0	0
Odontomachus rixosus	0	2	0	0	0	0	0	0	0
Odontomachus sp. 2	0	0	1	0	0	0	1	0	0
Odontomachus sp. 3	0	0	0	0	0	0	2	6	0
Subordinate Camponotini (SC)									
Camponotus sp. 1	0	0	0	0	1	0	0	0	0
Specialist Predator (SP)									
Leptogenys sp. 1	1	0	0	0	0	0	0	0	0
Odontoponera transversa	0	0	0	0	0	9	3	2	3
Pachycondyla sp. 1	0	0	0	0	0	1	0	0	0
Tropical-climate Specialists (TCS)									
Anoplolepis gracilipes	4	0	67	0	55	0	0	0	32
Euprenolepis sp. 1	7	20	0	0	0	0	0	0	0

Dolichoderus sp.1	0	11	0	0	0	0	0	0	0
Dolichoderus bituberculatus	0	141	0	0	0	0	0	0	0
Gnamptogenys sp. 1	0	0	0	0	0	0	2	1	4
